# Limited benefit of adjuvant chemotherapy for Asian patients with stage IB lung adenocarcinoma: implications for clinical practice

**DOI:** 10.1186/s12957-025-03907-x

**Published:** 2025-07-07

**Authors:** Shaowei Xin, Jie Li, Long Jiang, Jianfei Zhu, Jie Lei, Jinbo Zhao, Miaomiao Wen, Yahui Tian, Zitong Wan, Yujie Guo, Yinxi Zhou, Suxin Jiang, Chunlong Zheng, Yong Han, Yongfu Ma, Yanlu Xiong

**Affiliations:** 1 Department of Thoracic Surgery, Tangdu Hospital, Fourth Military Medical University, 569 Xinsi Road, Baqiao District, Xi’an City, Shaanxi Province 710038 China; 2 Department of Thoracic Surgery, Air Force Medical Center, Fourth Military Medical University, 30 Fucheng Road, Haidian District, Beijing City, 100142 China; 3 Department of Thoracic Surgery, The 962nd Hospital of the Chinese People’s Liberation Army Joint Logistics Support Force, Harbin, China; 4 Department of Pathology, First Medical Center, Chinese PLA General Hospital, Beijing, China; 5 Shanghai Lung Cancer Center, Shanghai Chest Hospital, Shanghai Jiao Tong University School of Medicine, Shanghai, China; 6 Department of Thoracic Surgery, Shaanxi Provincial People’s Hospital, Xi’an, Shaanxi China; 7 College of Life Sciences, Northwest University, Xi’an, Shaanxi China; 8 Department of Thoracic Surgery, First Medical Center, Chinese PLA General Hospital, 28 Fuxing Road, Haidian District, Beijing, 100853 China; 9 Innovation Center for Advanced Medicine, Tangdu Hospital, Fourth Military Medical University, Xi’an, China

**Keywords:** Lung adenocarcinoma, Adjuvant chemotherapy, Stage IB, SEER

## Abstract

**Backgroud:**

The benefit of adjuvant chemotherapy for patients with stage IB lung adenocarcinoma remains unclear. We compared adjuvant chemotherapy outcomes in Asian and non-Asian stage IB lung adenocarcinoma patients using multi-source clinical data.

**Methods:**

Patients with stage IB lung adenocarcinoma from the 2004–2015 Surveillance, Epidemiology, and End Results (SEER) database and the 2020–2021 thoracic surgery department of three Chinese medical centers were included. Patients were divided into adjuvant chemotherapy and observation groups. Propensity score matching (PSM) was performed to reduce confounding bias. Survival curves were plotted and compared using the Kaplan–Meier method and log-rank test, respectively.

**Results:**

In the SEER data, 3408 patients met the inclusion criteria, overall survival (OS) was significantly better in the adjuvant chemotherapy group than in the observation group. In the non-Asian population, OS was significantly better in the adjuvant chemotherapy group, both before and after PSM. In the Asian population, OS did not significantly differ between the two groups before or after PSM. Among 690 patients from the three centers, disease-free survival (DFS) and OS in the adjuvant chemotherapy group were not better than those in the observation group, before or after PSM and in patients with high pathological grade or visceral pleural infiltration (VPI).

**Conclusions:**

Adjuvant chemotherapy can significantly improve the prognosis of non-Asian patients with stage IB lung adenocarcinoma. In Asian patients, adjuvant chemotherapy does not improve prognosis, even in patients with high pathological grade or VPI.

**Supplementary Information:**

The online version contains supplementary material available at 10.1186/s12957-025-03907-x.

## Introduction

Lung cancer is the leading cause of cancer death worldwide in 2022, accounting for 18.7% of all cancer deaths, with non-small cell lung cancer (NSCLC) comprising approximately 85% of these cases [1–3]. Surgery is the predominant treatment for early-stage NSCLC; however, many patients still have a poor prognosis due to postoperative recurrence [4, 5]. Postoperative adjuvant therapy is crucial for improving prognosis [6]. 

A series of previous randomized controlled clinical trials established adjuvant chemotherapy (CT) as the standard of care after stage II-IIIA NSCLC but with only a 5% improvement in survival [7–9]. The Adjuvant Navelbine International Trialist Association study explored the survival impact of adjuvant vinorelbine plus cisplatin in completely resected stage IB-IIIA NSCLC, and no survival benefit was observed in patients with stage IB [7]. The Cancer and Leukemia Group B 9633 study explored the effect of paclitaxel/carboplatin-based adjuvant CT on stage IB NSCLC and confirmed that adjuvant CT provided survival benefit in patients with stage IB NSCLC having tumors > 4 cm in diameter but yielded no survival benefit in those with tumors < 4 cm in diameter [8]. Additionally, a retrospective analysis of data from the National Cancer Data Base (NCDB) revealed that adjuvant CT significantly improved overall survival (OS) in stage IB NSCLC with a diameter ≤ 4 cm [10]. The new 9th edition of the American Joint Committee on Cancer staging system follows the 8th edition which classifies tumors > 4 cm but ≤ 5 cm without lymph node metastases as stage IIA [11]. Therefore, whether patients with 9th edition stage IB NSCLC benefit from adjuvant CT remains controversial.

Several studies also suggest that the effectiveness of adjuvant CT for stage IB NSCLC varies across ethnic populations [12–14]. Previous studies have not distinguished between pathological subtypes of NSCLC, and different pathological subtypes of NSCLC have varying prognoses and sensitivities to adjuvant CT [15, 16]. Lung adenocarcinoma has become the most common pathological subtype of NSCLC due to changes in the disease spectrum of lung cancer; however, the benefit of adjuvant CT for stage IB lung adenocarcinoma remains unclear [1, 17]. This study aimed to evaluate the prognostic impact of adjuvant CT on the prognosis of patients with stage IB lung adenocarcinoma (9th edition) who underwent complete surgical resection in the Surveillance, Epidemiology, and End Results (SEER) database from 2004 to 2015 and in three medical centers in China from 2020 to 2021.

## Methods

### Data source

**Fig. 1:**
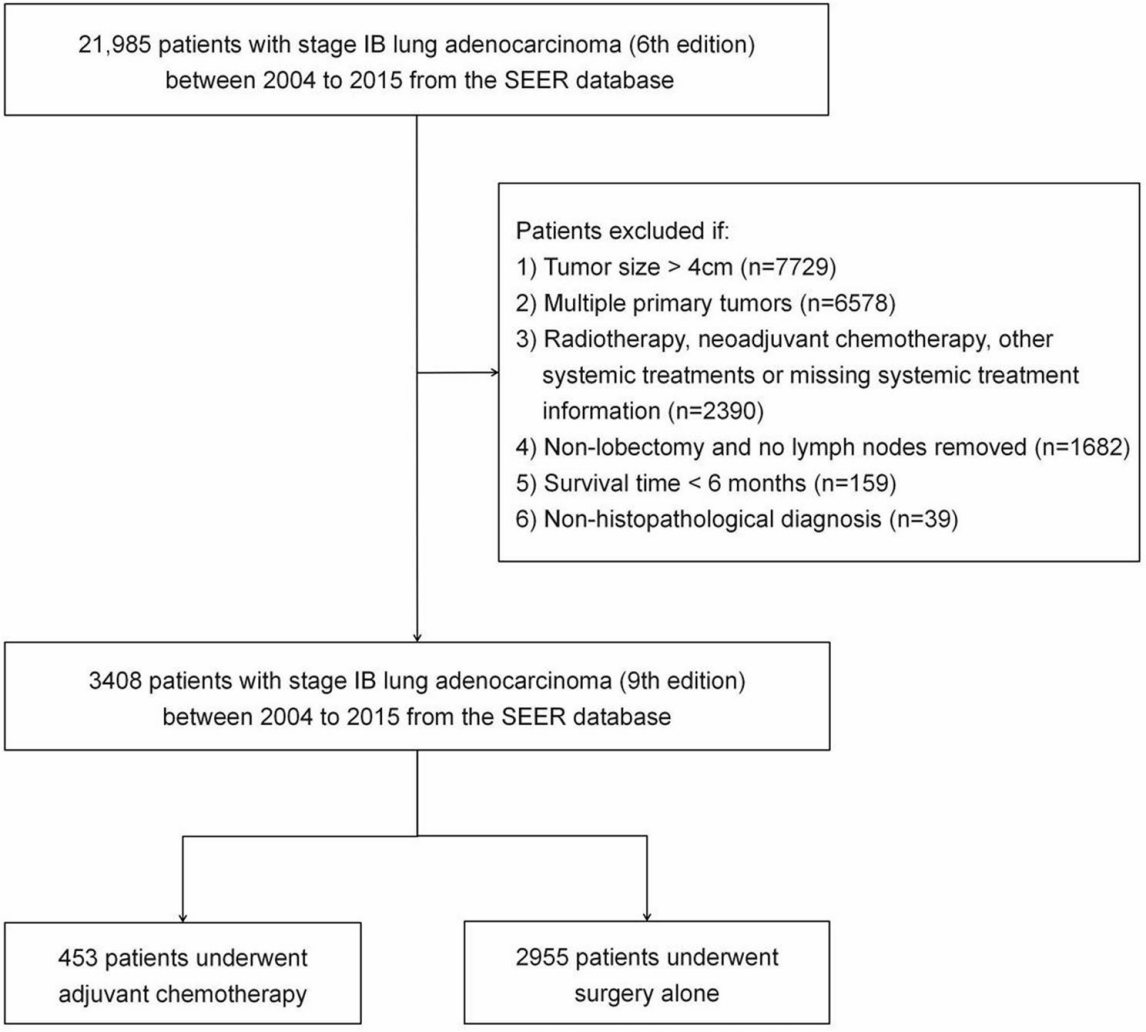
Patient inclusion flowchart. SEER, Surveillance, Epidemiology, and End Results

Using the SEER database (SEER*Stat 8.4.3 software, https://seer.cancer.gov/seerstat/software/), we selected patients with stage IB lung adenocarcinoma diagnosed between 2004 and 2015. Inclusion criteria were: (1) pathological diagnosis of lung adenocarcinoma, (2) T2aN0M0 stage tumor according to 9th edition TNM staging system classification (tumor diameter > 30 mm but ≤ 40 mm; invades visceral pleura; invades an adjacent lobe; involves main bronchus (up to but not including the carina) or is associated with atelectasis or obstructive pneumonitis, extending to the hilar region, involving either part of or the entire lung), and (3) radical lobectomy (R0 resection). Exclusion criteria were: (1) multiple primary or non-primary lung cancers, (2) lung wedge resection or sublobectomy, (3) absence of lymph node dissection, (4) other systemic therapies such as radiotherapy, targeted therapy, immunotherapy, image-guided thermal ablation therapy, (5) neoadjuvant CT, (6) death within 6 months, and (7) lack of necessary information. The inclusion process is shown in Fig. 1.

Our analysis included 690 patients with stage IB lung adenocarcinoma who underwent complete surgical resection at the thoracic surgery departments of three large medical centers in China (Tangdu Hospital of Air Force Military Medical University, Shanghai Chest Hospital, and First Medical Center of Chinese PLA General Hospital) during the period from January 2020 to December 2021. Only patients who underwent complete mediastinal lymph dissection were included, and the remaining inclusion and exclusion criteria were the same as above. All patients underwent at least four cycles of platinum-containing two-drug chemotherapy after surgery. Routine follow-up, including physical examination, hematological tests, and chest CT, was performed at 3–6 month intervals for the first 2 years and subsequently, at 6-month intervals. All patient information was anonymized and de-identified. As this is a retrospective study, informed consent was waived and approved by the ethics committee (K202402-41).

### Variable definitions

Patients were divided into adjuvant CT and observation groups (surgery only) based on whether or not they received postoperative adjuvant CT. In the SEER database subgroup analysis, we defined patients of Asian ethnicity (Non-hispanic Asian or Pacific Islander were selected from race and ancestry codes and Hawaiian, Pacific Islander and Samoan were excluded from race/ethnicity) as the Asian group and others as the non-Asian group. The category of poorly differentiated tumors includes grade III (poorly differentiated) and grade IV (undifferentiated/anaplastic). Visceral pleural invasion (VPI) is defined as as tumor penetration beyond the elastic layer (PL1) or extension to the visceral pleural surface (PL2), consistent with IASLC staging [18]. The solid/micropapillary subtypes are defined as tumors in which the solid or micropapillary component constitutes the predominant histologic pattern, according to the 2015 WHO classification [19]. OS was defined as the time interval from surgery to death or the last follow-up. DFS was defined as the time interval from the surgery to recurrence, death, or the last follow-up.

### Statistical analysis

Statistical analyses were performed using SPSS version 23.0 (IBM Corp., Armonk, NY). Pearson’s chi-square test was used to compare the demographic, clinical, and pathological characteristics of adjuvant CT and observation groups. Survival curves were plotted and propensity score matching (PSM) of the data was performed using R 4.3.3 statistical software. The Kaplan–Meier method and log-rank test were used to plot and compare OS and DFS survival curves. PSM was performed using the MatchIt software package to balance the baseline characteristics of adjuvant CT and observation groups. Multivariable Cox regression analysis was performed to identify independent risk factors. After calculating the propensity score using logistic regression, nearest-neighbor matching was performed using a caliper width set to 0.02 standard deviation. Statistical significance was set at *P* < 0.05.

## Results

### Survival analysis of patients in the SEER database cohort

**Fig. 2:**
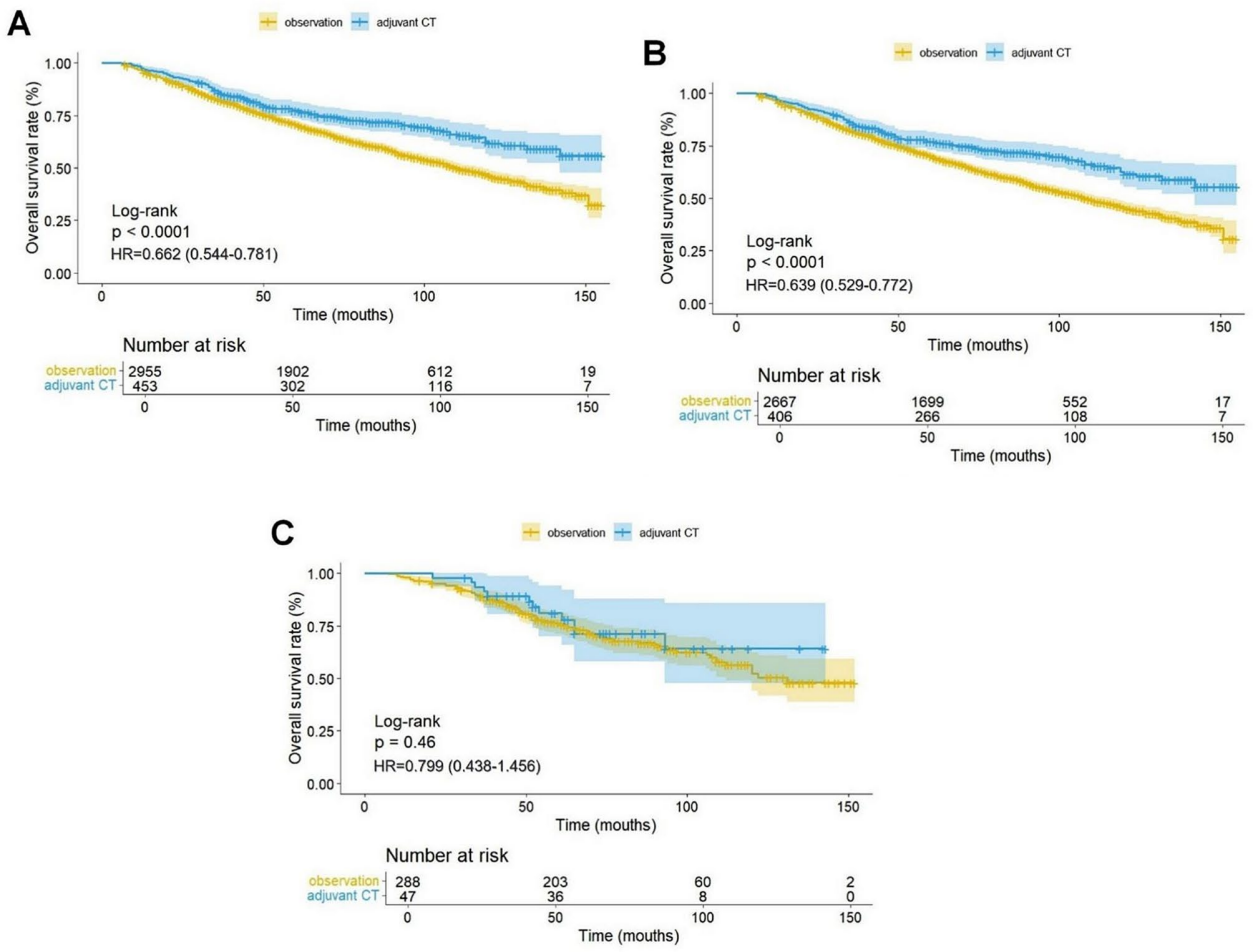
Kaplan-Meier curves for overall survival of the overall cohort (**A**), the non-Asian patients (**B**), the Asian patients (**C**). CT, chemotherapy; HR, hazard ratio

**Fig. 3:**
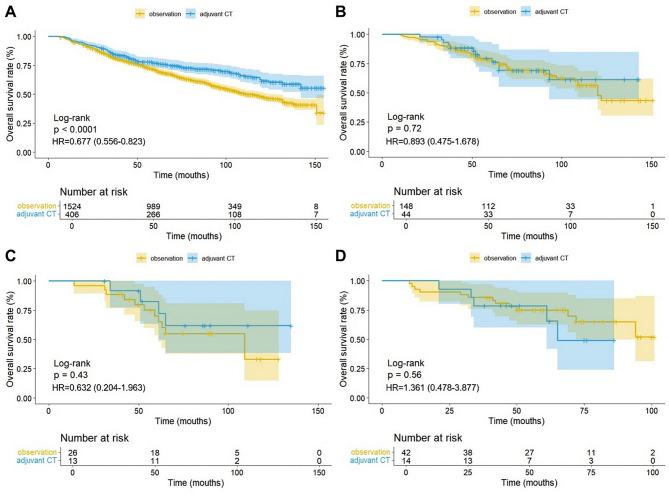
Kaplan-Meier curves for overall survival of the non-Asian patients (**A**), the Asian patients (**B**), the Asian patients with poorly differentiated (**C**), and the Asian patients with VPI (**D**) after propensity score matching. CT, chemotherapy; VPI, visceral pleural infiltration; HR, hazard ratio

A total of 3408 patients with stage IB lung adenocarcinoma were included from the SEER database (Table 1). Data for the non-Asian and Asian groups are shown in supplementary Tables 1 and supplementary Table 2. In the entire cohort, the adjuvant CT group had better OS than the observation group (HR, 0.662; 95% CI, 0.544–0.781; *P* < 0.0001) (Fig. 2A). In the non-Asian population, the adjuvant CT group had significantly better OS than the observation group (HR, 0.639; 95% CI, 0.529–0.772; *P* < 0.0001) (Fig. 2B). However, in the Asian population, there was no statistical difference in OS between the two groups (HR, 0.799; 95% CI, 0.438–1.456; *P* = 0.46) (Fig. 2C). After PSM, in the non-Asian population, the OS of the adjuvant CT group remained significantly better than that of the observation group (HR, 0.677; 95% CI, 0.556–0.823; *P* < 0.0001) (Fig. 3A). There was still no statistical difference in OS between the adjuvant CT and observation groups in the Asian population after PSM (HR, 0.893; 95% CI, 0.475–1.678; *P* = 0.72) (Fig. 3B). Subgroup analysis revealed no statistical difference in OS between the adjuvant CT and observation groups for patients with poorly differentiated adenocarcinoma (HR, 0.632; 95% CI, 0.204–1.963; *P* = 0.43) or those with VPI (HR, 1.361; 95% CI, 0.478–3.877; *P* = 0.56) (Fig. 3C, D). We have reconfirmed this subgroup analysis result in the data between PSM (Supplementary Fig. 1).

**Table 1:** Baseline characteristics of 3048 patients with stage IB lung

Characteristics	Total (*n* = 3408)	Adjuvant CT (*n* = 453)	Observation (*n* = 2955)	*P* value
Sex, n (%) Female Male	1992 (58.5) 1416 (41.5)	269 (59.4) 184 (40.6)	1723 (58.3) 1232 (41.7)	0.666
Age, n (%) ≤ 60 y > 60 y	916 (26.9) 2492 (73.1)	193 (42.6) 260 (57.4)	723 (24.5) 2232 (75.5)	< 0.001
Race, n (%) Asian Non-Asian	335 (9.8) 3073 (90.2)	47 (10.4) 406 (89.6)	288 (9.7) 2667 (90.3)	0.675
Tumor size, n (%) ≤ 3 cm > 3 cm	1722 (50.5) 1686 (49.5)	204 (45.0) 249 (55.0)	1518 (51.4) 1437 (48.6)	0.012
Laterality, n (%) Left Right	1336 (39.2) 2072 (60.8)	179 (39.5) 274 (60.5)	1157 (39.2) 1798 (60.8)	0.884
Grade, n (%) Well and Moderately Poorly Undifferentiated	2346 (68.8) 932 (27.3) 130 (3.8)	279 (61.6) 164 (36.2) 10 (2.2)	2067 (69.9) 768 (26.0) 120 (4.1)	< 0.001
VPI, n (%) No Yes Undifferentiated	860 (25.3) 631 (18.5) 1917 (56.3)	93 (20.5) 94 (20.8) 266 (58.7)	767 (25.9) 537 (18.2) 1651 (55.9)	0.038
LNs examined, n (%) 1–3 4–6 > 6	526 (15.4) 811 (23.8) 2071 (60.8)	74 (16.3) 109 (24.1) 270 (59.6)	452 (15.3) 702 (23.8) 1801 (60.9)	0.816

CT, chemotherapy; VPI, visceral pleural infiltration; LN, lymph node

### Patient characteristics from multiple centers

A total of 690 patients from the Department of Thoracic Surgery at three medical centers were enrolled, including 247 from Tangdu Hospital of Air Force Military Medical University, 242 from Shanghai Chest Hospital, and 201 from the First Medical Center of Chinese PLA General Hospital. There were 245 (35.5%) patients in the adjuvant CT group and 445 (64.5%) in the observation group (Table 2). After 1:1 PSM, 200 pairs of patients were successfully matched (Supplementary Table 3).

**Table 2:** Baseline characteristics of 690 patients with stage IB lung adenocarcinoma

Characteristic	All patients (*n* = 690)
Age > 60y, n (%)	408 (59.1)
Male sex, n (%)	324 (47.0)
Smoking history, n (%)	168 (24.3)
Tumor diameter > 3 cm, n (%)	434 (62.9)
GGN, n (%)	434 (62.9)
Tumor location, n (%) RUL RML RLL LUL LLL	248 (35.9) 46 (6.7) 111 (16.1) 185 (26.8) 100 (14.5)
Solid/micropapillary subtype, n (%)	61 (8.8)
IASLC grade, n (%) Grade 1 Grade 2 Grade 3	61 (8.8) 498 (72.2) 131 (19.0)
VPI, n (%)	332 (48.1)
Adjuvant CT, n (%)	245 (35.5)

GGN, ground-glass nodule; RUL, right upper lobe; RML, right middle lobe; RLL, right lower lobe; LUL, left upper lobe; LLL, left lower lobe; IASLC, International Association for the Study of Lung Cancer; VPI, visceral pleural infiltration; CT, chemotherapy

### Survival analysis of multicenter patients

**Fig. 4:**
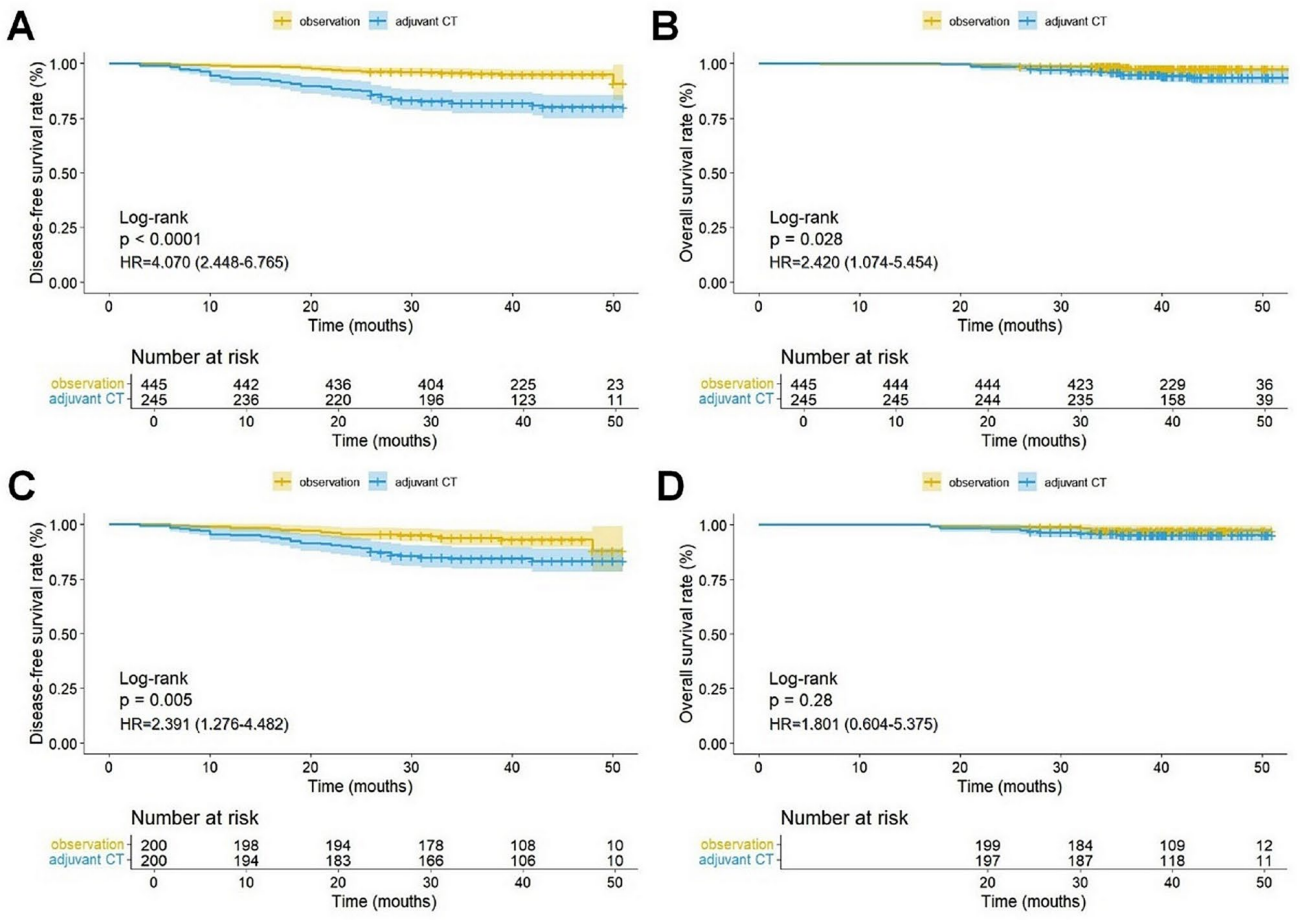
Kaplan-Meier curves of the multicenter data for disease-free survival (**A**), overall survival (**B**) before propensity score matching and disease-free survival (**C**), overall survival (**D**) after propensity score matching. CT, chemotherapy; HR, hazard ratio

**Fig. 5:**
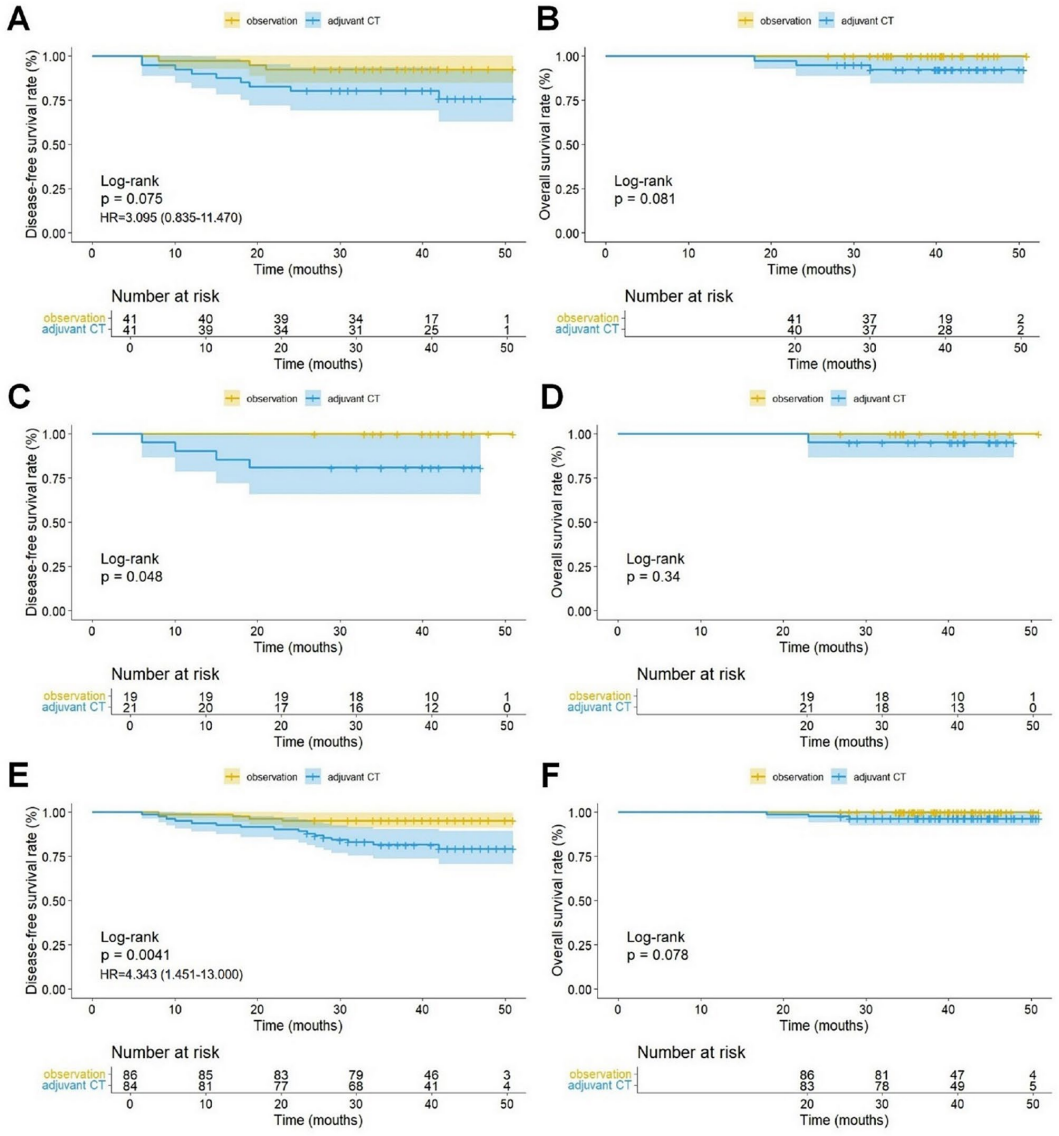
Kaplan-Meier curves of the multicenter data for disease-free survival (**A**), overall survival (**B**) in patients with IASLC grade 3, disease-free survival (**C**), overall survival (**D**) in patients with solid/micropapillary subtypes and disease-free survival (**E**), overall survival (**F**) in patients with VPI. CT, chemotherapy; IASLC, International Association for the Study of Lung Cancer; VPI, visceral pleural infiltration; HR, hazard ratio. The number of events in **B**, **C**, **D**, and **F** was insufficient to provide reliable HRs

Before PSM, DFS (HR, 4.070; 95% CI, 2.448–6.765; *P* < 0.0001) and OS (HR, 2.420; 95% CI, 1.074–5.454; *P* = 0.028) in the adjuvant CT group were significantly worse than those in the observation group (Fig. 4A, B). After PSM, the adjuvant CT group still had worse DFS than the observation group (HR, 2.391; 95% CI, 1.276–4.482; *P* = 0.005); however, no significant difference in OS was observed (HR, 1.801; 95% CI, 0.604–5.375; *P* = 0.28) (Fig. 4C, D). Subgroup analysis revealed that in patients with IASLC grade 3, DFS (*P* = 0.075) and OS (*P* = 0.081) in the adjuvant CT group were not significantly different from those in the observation group (Fig. 5A, B). Among patients with solid/micropapillary subtypes, DFS in the adjuvant CT group was significantly worse than that in the observation group (*P* = 0.048); however, there was no significant difference in OS (*P* = 0.34) (Fig. 5C, D). Additionally, in patients with VPI, DFS in the adjuvant CT group was significantly worse than that in the observation group (*P* = 0.0041), while OS showed no significant difference (*P* = 0.078) (Fig. 5E, F). The subgroup analysis results were confirmed in the data before PSM (Supplementary Fig. 2).

### Multivariate Cox regression analysis of OS and DFS

In the SEER database, multivariable Cox regression analysis of the non-Asian population demonstrated that adjuvant CT was an independent protective factor for overall survival (OS) in stage IB lung adenocarcinoma (HR = 0.695, 95% CI: 0.580–0.834, *P* < 0.001; Supplementary Table 4). In contrast, analysis of the Asian population revealed no significant association between adjuvant CT and OS benefit, as it was not an independent predictive factor (HR = 0.913, 95% CI: 0.498–1.674, *P* = 0.768; Supplementary Table 5). In the multi-center cohort, adjuvant CT was identified as an independent risk factor for inferior DFS in stage IB lung adenocarcinoma (HR = 3.679, 95% CI: 2.207–6.133, *P* < 0.001; Supplementary Table 6). In the SEER Asian population data, adjuvant CT was not an independent prognostic factor for OS in either the poorly differentiated subgroup (HR, 0.478; 95% CI, 0.153–1.490; *P* = 0.203; Supplementary Table 7) or the VPI subgroup (HR, 1.015; 95% CI, 0.345–2.983; *P* = 0.979; Supplementary Table 8). In contrast, the multi-center dataset subgroup analysis demonstrated that adjuvant CT served as an independent risk factor for DFS in both the IASLC grade 3 subgroup (HR, 4.749; 95% CI, 1.543–14.614; *P* = 0.007; Supplementary Table 9) and the VPI subgroup (HR, 4.947; 95% CI, 2.376–10.302; *P* < 0.001; Supplementary Table 10). A similar trend toward increased risk was observed in the solid/micropapillary subtypes, though it did not reach statistical significance (HR, 6.658; 95% CI, 0.770-57.559; *P* = 0.085; Supplementary Table 11). In subgroup analyses of the multi-center data, adjuvant CT also failed to reduce mortality risk (Supplementary Tables 12, 13, 14).

## Discussion

Lung adenocarcinoma has surpassed lung squamous cell carcinoma as the most common histological type of lung cancer, with an increasing incidence [17, 20]. A retrospective study showed that the median OS in the adjuvant CT group was significantly better than that in the observation group (75.0 vs. 57.4 months, *P* < 0.001), and the median treatment failure-free survival (TFS) was also better than that in the observation group for early-stage lung squamous cell carcinoma [21]. However, the median OS and median TFS time were not significantly different between the adjuvant CT and observation groups in patients with lung adenocarcinoma, suggesting that lung adenocarcinoma was less sensitive to CT [21]. However, our analysis of the SEER database revealed that adjuvant CT significantly improved the OS of stage IB lung adenocarcinoma.

Race may be another important factor affecting the outcome of adjuvant CT. A retrospective study of 569 patients with NSCLC from China revealed that 5-year OS was worse in the adjuvant CT group than in the observation group (82.4% vs. 87.6%, *P* = 0.021).^22^ A large retrospective analysis in Japan found no OS benefit in the adjuvant CT group compared to the observation group for stage IB NSCLC [23]. A retrospective analysis of NCDB data found that for stage IB NSCLC with tumor diameters of 3.1–3.9 cm, the median and 5-year OS significantly improved from 78.9 months and 60% in the observation group to 101.6 months and 68% in the adjuvant CT group, respectively [10]. A multivariate analysis of OS in 9757 patients with stage IB NSCLC from SEER data revealed that other races (such as Indian and Asian) had a better prognosis than white patients, with no significant difference in outcome between the black and white patients [13]. Only white patients showed a better benefit from adjuvant CT after PSM, although the difference was not statistically significant. These studies further suggest the influence of race on adjuvant CT outcomes. In the present study, after grouping the SEER database data based on Asian and non-Asian populations, subgroup analysis showed that non-Asian patients exhibited significantly improved OS regardless of adjuvant CT before and after PSM; however, in Asian patients, there was no significant difference in OS between the two groups before and after PSM. Multivariate COX regression further confirmed these results.

The observed racial disparities in treatment efficacy likely arise from multifactorial and complex mechanisms. Asian patients with stage IB lung adenocarcinoma are predominantly Epidermal growth factor receptor (EGFR) mutated and exhibit better prognosis, whereas Caucasian populations show higher prevalence of Kirsten rat sarcoma viral oncogene homolog (K-ras) mutations [24–26]. However, differences in driver mutations may not be the primary determinant of CT efficacy, as multiple studies indicate that adjuvant CT outcomes remain independent of oncogenic mutation status [27, 28]. Additionally, the clinical trials laying the foundation for adjuvant CT are mainly based on white populations, and there may be differences in the dose, response, and tolerance of adjuvant CT between Asian and white populations [7, 8]. Asian patients undergoing platinum-based doublet CT regimens experience more severe toxic side effects, leading to dose reductions aimed at improving treatment adherence and completion rates, which may inadvertently result in subtherapeutic dosing [29]. Moreover, the higher genomic instability observed in Asian populations leads to the presence of multiple genetically distinct subclones and significant intratumoral heterogeneity, thereby reducing therapeutic efficacy [30, 31]. Studies have revealed that ERCC1 genetic polymorphisms significantly reduce sensitivity to platinum-based CT and shorten overall survival in Asian populations, whereas no such effects are observed in Caucasian populations [32, 33]. Similarly, RRM1 polymorphisms correlate with CT response exclusively in Asian cohorts, underscoring race-specific disparities in chemotherapeutic efficacy [34]. These emphasize that ethnicity-stratified treatment protocols are essential for optimizing lung cancer outcomes and exploring the underlying biological mechanisms further.

To exclude the influence of sublobectomy and lymph node resection status on our results, we only included patients who underwent complete lobectomy with mediastinal lymph node dissection in our multicenter study. Our findings revealed that adjuvant CT resulted in worse PFS for stage IB lung adenocarcinoma before and after PSM. The OS of the adjuvant CT group was worse than that of the observation group before PSM, and no significant difference was observed between the two groups after PSM. These findings are consistent with previous reports on NSCLC [22]. 

Stage IB lung adenocarcinoma exhibits extensive heterogeneity, and some tumor-specific variables are useful for distinguishing prognosis [35]. VPI is associated with a poor prognosis in NSCLC; however, whether it can guide chemotherapy remains controversial. [36, 37] Xie et al. [36, 37] found that patients with stage IB NSCLC and VPI did not benefit from adjuvant CT, whereas Wightman et al. [29] reported the opposite. This discrepancy may be related to the heterogeneity of the population and pathological subtypes in the study. Our study showed that adjuvant CT significantly improved OS in non-Asian patients with VPI; however, there was no difference in OS between the adjuvant CT and the observation group in Asian patients with VPI. Moreover, our multicenter data show that adjuvant CT leads to worse PFS even with VPI. Multicenter analysis further demonstrated that adjuvant CT failed to improve prognosis in IASLC grade 3 stage IB lung adenocarcinoma, with similar outcomes observed in solid/micropapillary subtypes. However, non-Asian populations derived survival benefits from adjuvant CT regardless of differentiation status. Multivariable analyses confirmed all these findings. While these findings enhance the robustness of our conclusions, it is important to note that the limited sample size in certain subgroups resulted in wide confidence intervals. To avoid overinterpretation, future efforts should focus on expanding the sample size or validating these results in prospective studies.

Emerging evidence has revealed the promise of targeted and immunotherapies in adjuvant treatment for stage IB lung cancer. The CORIN trial demonstrated a 3-year DFS rate of 96.1% in the icotinib group versus 84.0% in the observation group (HR = 0.23), though the small sample size (*n* = 128) necessitates larger phase III validation [38]. The 5-year OS results of the ADAURA trial showed that osimertinib achieved significant DFS and OS improvements in the stage IB subgroup, with a 4-year DFS rate of 80% (vs. 59% with placebo; HR = 0.44) and a 5-year OS rate of 94% (vs. 88% with placebo; HR = 0.44), reducing mortality risk by 56% [39]. But the study used the 7th edition of the staging system, so the therapeutic effect on IB stage (tumors < 4 cm) remains unclear. In the IMpower010 trial, adjuvant atezolizumab significantly prolonged DFS, particularly in patients with PD-L1 expression ≥ 1% (HR = 0.66), leading to its approval for adjuvant treatment in PD-L1 ≥ 1% stage II-IIIA NSCLC [40]. The KEYNOTE-091 trial demonstrated DFS benefits with adjuvant pembrolizumab independent of PD-L1 expression [41]. However, neither trial reported separate stage IB subgroup data. These studies demonstrate the efficacy of targeted therapy and immunotherapy in specific cohorts of stage IB lung cancer patients, highlighting the heterogeneity of this disease stage. Further exploration is required to establish optimal criteria for determining the necessity of adjuvant therapy and selecting appropriate treatment modalities for stage IB lung adenocarcinoma.

This study has certain limitations. First, while our multicenter data implemented strict inclusion criteria for CT, the SEER database lacks detailed CT information. Second, although the therapeutic efficacy of CT agents and tumor genetic backgrounds remained largely unchanged, the temporal disparity between the SEER and multicenter datasets introduced additional complexity to the results. Third, there was no long-term follow-up of multicenter data. Finally, this retrospective study requires further validation through large-scale prospective clinical trials.

In summary, our data suggest that adjuvant CT significantly improved outcomes in non-Asian patients with stage IB lung adenocarcinoma. However, for patients with stage IB lung adenocarcinoma in the Asian population, adjuvant CT did not improve prognosis and is not recommended, even in patients with high pathologic grade or VPI.

## Electronic supplementary material

Below is the link to the electronic supplementary material.

Supplementary Material 1

Supplementary Material 2

## Data Availability

No datasets were generated or analysed during the current study.

## Abbreviations

SEER: Surveillance, Epidemiology, and End Results
PSM: Propensity Score Matching
OS: Overall Survival
DFS: Disease-Free Survival
VPI: Visceral Pleural Infiltration
NSCLC: Non-Small Cell Lung Cancer
CT: Chemotherapy
IASLC: International Association for the Study of Lung Cancer
TFS: Treatment failure-free survival
EGFR: Epidermal Growth Factor Receptor
K-ras: Kirsten rat sarcoma viral oncogene homolog

## References

[1] Bray F, Laversanne M, Sung H, et al. Global cancer statistics 2022: GLOBOCAN estimates of incidence and mortality worldwide for 36 cancers in 185 countries. CA Cancer J Clin. 2024;74(3):229–63. 38572751 10.3322/caac.21834

[2] Luo G, Zhang Y, Rumgay H, et al. Estimated worldwide variation and trends in incidence of lung cancer by histological subtype in 2022 and over time: a population-based study. Lancet Respir Med. 2025;13(4):348–63. 39914442 10.1016/S2213-2600(24)00428-4

[3] Liu X, Xi X, Xu S, et al. Targeting T cell exhaustion: emerging strategies in non-small cell lung cancer. Front Immunol. 2024;15:1507501. 39726592 10.3389/fimmu.2024.1507501 PMC11669709

[4] Zhang P, Duan J, Bai H, et al. Influence of adjuvant chemotherapy on survival for patients with stage IB and IIA non-small cell lung cancer. Thorac Cancer. 2021;12(1):30–9. 33111432 10.1111/1759-7714.13685 PMC7779205

[5] Siegel RL, Giaquinto AN, Jemal A. Cancer statistics, 2024. CA Cancer J Clin. 2024;74(1):12–49. 10.3322/caac.21820 10.3322/caac.21820 38230766

[6] Shen ZQ, Feng KP, Fang ZY, et al. Influence of adjuvant chemotherapy on survival for patients with completely resected high-risk stage IB NSCLC. J Cardiothorac Surg. 2024;19(1):1. 38166960 10.1186/s13019-023-02457-1 PMC10763355

[7] Douillard JY, Rosell R, De Lena M, et al. Adjuvant Vinorelbine plus cisplatin versus observation in patients with completely resected stage IB-IIIA non-small-cell lung cancer (Adjuvant navelbine international trialist association [ANITA]): a randomised controlled trial. Lancet Oncol. 2006;7(9):719–27. 16945766 10.1016/S1470-2045(06)70804-X

[8] Strauss GM, Herndon JE 2nd, Maddaus MA, et al. Adjuvant Paclitaxel plus carboplatin compared with observation in stage IB non-small-cell lung cancer: CALGB 9633 with the Cancer and leukemia group B, radiation therapy oncology group, and North central Cancer treatment group study groups. J Clin Oncol. 2008;26(31):5043–51. 10.1200/JCO.2008.16.4855 PMC2652093 18809614

[9] Douillard JY, Tribodet H, Aubert D, et al. Adjuvant cisplatin and Vinorelbine for completely resected non-small cell lung cancer: subgroup analysis of the lung adjuvant cisplatin evaluation. J Thorac Oncol. 2010;5(2):220–8. 20027124 10.1097/JTO.0b013e3181c814e7

[10] Morgensztern D, Du L, Waqar SN, et al. Adjuvant chemotherapy for patients with T2N0M0 NSCLC. J Thorac Oncol. 2016;11(10):1729–35. 27287414 10.1016/j.jtho.2016.05.022 PMC5141602

[11] Rami-Porta R, Nishimura KK, Giroux DJ, et al. The international association for the study of lung Cancer lung Cancer staging project: proposals for revision of the TNM stage groups in the forthcoming (Ninth) edition of the TNM classification for lung Cancer. J Thorac Oncol. 2024;19(7):1007–27. 38447919 10.1016/j.jtho.2024.02.011

[12] Liang W, Zhong R, He J. Osimertinib in EGFR-Mutated lung Cancer. N Engl J Med. 2021;384(7):675. 33596364 10.1056/NEJMc2033951

[13] Xu Y, Wan B, Zhu S, et al. Effect of adjuvant chemotherapy on survival of patients with 8th edition stage IB Non-Small cell lung Cancer. Front Oncol. 2021;11:784289. 35155190 10.3389/fonc.2021.784289 PMC8828472

[14] Park HJ, Park HS, Cha YJ, et al. Efficacy of adjuvant chemotherapy for completely resected stage IB non-small cell lung cancer: a retrospective study. J Thorac Dis. 2018;10(4):2279–87. 29850132 10.21037/jtd.2018.03.184 PMC5949474

[15] Soh J, Toyooka S, Okumura N, et al. Impact of pathological stage and histological subtype on clinical outcome of adjuvant chemotherapy of Paclitaxel plus carboplatin versus oral uracil-tegafur for non-small cell lung cancer: subanalysis of SLCG0401 trial. Int J Clin Oncol. 2019;24(11):1367–76. 31312931 10.1007/s10147-019-01508-9

[16] Wang BY, Huang JY, Chen HC, et al. The comparison between adenocarcinoma and squamous cell carcinoma in lung cancer patients. J Cancer Res Clin Oncol. 2020;146(1):43–52. 31705294 10.1007/s00432-019-03079-8 PMC11804334

[17] Succony L, Rassl DM, Barker AP, McCaughan FM, Rintoul RC. Adenocarcinoma spectrum lesions of the lung: detection, pathology and treatment strategies. Cancer Treat Rev. 2021;99:102237. 34182217 10.1016/j.ctrv.2021.102237

[18] Travis WD, Brambilla E, Rami-Porta R, et al. Visceral pleural invasion: pathologic criteria and use of elastic stains: proposal for the 7th edition of the TNM classification for lung cancer. J Thorac Oncol. 2008;3(12):1384–90. 19057261 10.1097/JTO.0b013e31818e0d9f

[19] Travis WD, Brambilla E, Nicholson AG, et al. The 2015 world health organization classification of lung tumors: impact of genetic, clinical and radiologic advances since the 2004 classification. J Thorac Oncol. 2015;10(9):1243–60. 26291008 10.1097/JTO.0000000000000630

[20] Barta JA, Powell CA, Wisnivesky JP. Global Epidemiology of Lung Cancer. Ann Glob Health. 2019;85(1):8. Published 2019 Jan 22. 10.5334/aogh.2419 10.5334/aogh.2419 PMC6724220 30741509

[21] Hsiao SH, Chen WT, Chung CL, et al. Comparative survival analysis of platinum-based adjuvant chemotherapy for early-stage squamous cell carcinoma and adenocarcinoma of the lung. Cancer Med. 2022;11(10):2067–78. 35274494 10.1002/cam4.4570 PMC9119352

[22] Wang J, Wu N, Lv C, Yan S, Yang Y. Should patients with stage IB non-small cell lung cancer receive adjuvant chemotherapy? A comparison of survival between the 8th and 7th editions of the AJCC TNM staging system for stage IB patients. J Cancer Res Clin Oncol. 2019;145(2):463–9. 30474757 10.1007/s00432-018-2801-7 PMC11810431

[23] Adachi H, Saito A, Shintani Y, et al. Is adjuvant chemotherapy for completely resected p-stage IA (> 2 cm) and stage IB non-small-cell lung cancer beneficial for elderly patients? A large, retrospective cohort study based on real-world data from Japan. Jpn J Clin Oncol. 2023;53(12):1191–200. 37626449 10.1093/jjco/hyad116

[24] Zhao M, Zhan C, Li M, et al. Aberrant status and clinicopathologic characteristic associations of 11 target genes in 1,321 Chinese patients with lung adenocarcinoma. J Thorac Dis. 2018;10(1):398–407. 29600072 10.21037/jtd.2017.12.68 PMC5863124

[25] Rekhtman N, Ang DC, Riely GJ, Ladanyi M, Moreira AL. KRAS mutations are associated with solid growth pattern and tumor-infiltrating leukocytes in lung adenocarcinoma. Mod Pathol. 2013;26(10):1307–19. 23619604 10.1038/modpathol.2013.74 PMC3732528

[26] Xin S, Wen M, Tian Y, et al. Impact of histopathological subtypes on invasive lung adenocarcinoma: from epidemiology to tumour microenvironment to therapeutic strategies. World J Surg Oncol. 2025;23(1):66. 40016762 10.1186/s12957-025-03701-9 PMC11866629

[27] Lee KH, Han SW, Hwang PG, et al. Epidermal growth factor receptor mutations and response to chemotherapy in patients with non-small-cell lung cancer. Jpn J Clin Oncol. 2006;36(6):344–50. 16818479 10.1093/jjco/hyl039

[28] Park JH, Lee SH, Keam B, et al. EGFR mutations as a predictive marker of cytotoxic chemotherapy. Lung Cancer. 2012;77(2):433–7. 22521649 10.1016/j.lungcan.2012.03.020

[29] Sekine I, Yamamoto N, Nishio K, Saijo N. Emerging ethnic differences in lung cancer therapy. Br J Cancer. 2008;99(11):1757–62. 18985035 10.1038/sj.bjc.6604721 PMC2600690

[30] Chen JB, Yang HC, Teo ASM, et al. Genomic landscape of lung adenocarcinoma in East Asians. Nat Genet. 2020;52(2):177–86. 32015526 10.1038/s41588-019-0569-6

[31] Leppä AM, Grimes K, Jeong H, et al. Single-cell multiomics analysis reveals dynamic clonal evolution and targetable phenotypes in acute myeloid leukemia with complex karyotype. Nat Genet. 2024;56(12):2790–803. 39587361 10.1038/s41588-024-01999-x PMC11631769

[32] Tan LM, Qiu CF, Zhu T, et al. Genetic polymorphisms and Platinum-based chemotherapy treatment outcomes in patients with Non-Small cell lung cancer: A genetic epidemiology study based Meta-analysis. Sci Rep. 2017;7(1):5593. 28717179 10.1038/s41598-017-05642-0 PMC5514117

[33] Zhang Y, Cao S, Zhuang C, et al. ERCC1 rs11615 polymorphism and chemosensitivity to platinum drugs in patients with ovarian cancer: a systematic review and meta-analysis. J Ovarian Res. 2021;14(1):80. Published 2021 Jun 21. 34148553 10.1186/s13048-021-00831-y PMC8215742

[34] Ludovini V, Floriani I, Pistola L, et al. Association of cytidine deaminase and xeroderma pigmentosum group D polymorphisms with response, toxicity, and survival in cisplatin/gemcitabine-treated advanced non-small cell lung cancer patients. J Thorac Oncol. 2011;6(12):2018–26. 22052224 10.1097/JTO.0b013e3182307e1f

[35] Kris MG, Gaspar LE, Chaft JE, et al. Adjuvant systemic therapy and adjuvant radiation therapy for stage I to IIIA completely resected Non-Small-Cell lung cancers: American society of clinical oncology/cancer care Ontario clinical practice guideline update. J Clin Oncol. 2017;35(25):2960–74. 28437162 10.1200/JCO.2017.72.4401

[36] Xie J, Zhang X, Hu S, et al. Effects of adjuvant chemotherapy on survival of patients with stage IB non-small cell lung cancer with visceral pleural invasion. J Cancer Res Clin Oncol. 2020;146(9):2231–9. 32533405 10.1007/s00432-020-03276-w PMC11804489

[37] Wightman SC, Lee JY, Ding L, et al. Adjuvant chemotherapy for visceral pleural invasion in 3-4-cm non-small-cell lung cancer improves survival. Eur J Cardiothorac Surg. 2022;62(1):ezab498. 10.1093/ejcts/ezab498 10.1093/ejcts/ezab498 35325098

[38] Ou W, Li N, Wang BX, et al. Adjuvant Icotinib versus observation in patients with completely resected EGFR-mutated stage IB NSCLC (GASTO1003, CORIN): a randomised, open-label, phase 2 trial. EClinicalMedicine. 2023;57:101839. 36816343 10.1016/j.eclinm.2023.101839 PMC9932314

[39] Tsuboi M, Herbst RS, John T, et al. Overall survival with osimertinib in resected EGFR-Mutated NSCLC. N Engl J Med. 2023;389(2):137–47. 37272535 10.1056/NEJMoa2304594

[40] Felip E, Altorki N, Zhou C, et al. Adjuvant Atezolizumab after adjuvant chemotherapy in resected stage IB-IIIA non-small-cell lung cancer (IMpower010): a randomised, multicentre, open-label, phase 3 trial. Lancet. 2021;398(10308):1344–57. 34555333 10.1016/S0140-6736(21)02098-5

[41] O’Brien M, Paz-Ares L, Marreaud S, et al. Pembrolizumab versus placebo as adjuvant therapy for completely resected stage IB-IIIA non-small-cell lung cancer (PEARLS/KEYNOTE-091): an interim analysis of a randomised, triple-blind, phase 3 trial. Lancet Oncol. 2022;23(10):1274–86. 36108662 10.1016/S1470-2045(22)00518-6

