# Supplementary material for: Limited benefit of adjuvant chemotherapy for Asian patients with stage IB lung adenocarcinoma: implications for clinical practice
Source: World J Surg Oncol. 2025 Jul 7;23:267. doi: 10.1186/s12957-025-03907-x (PMC12236002; doi:10.1186/s12957-025-03907-x)

Supplementary Figure 1. Kaplan-Meier curves for overall survival of the Asian patients with poorly differentiated (A), and the Asian patients with VPI (B) before propensity score matching. CT, chemotherapy, VPI, visceral pleural infiltration.

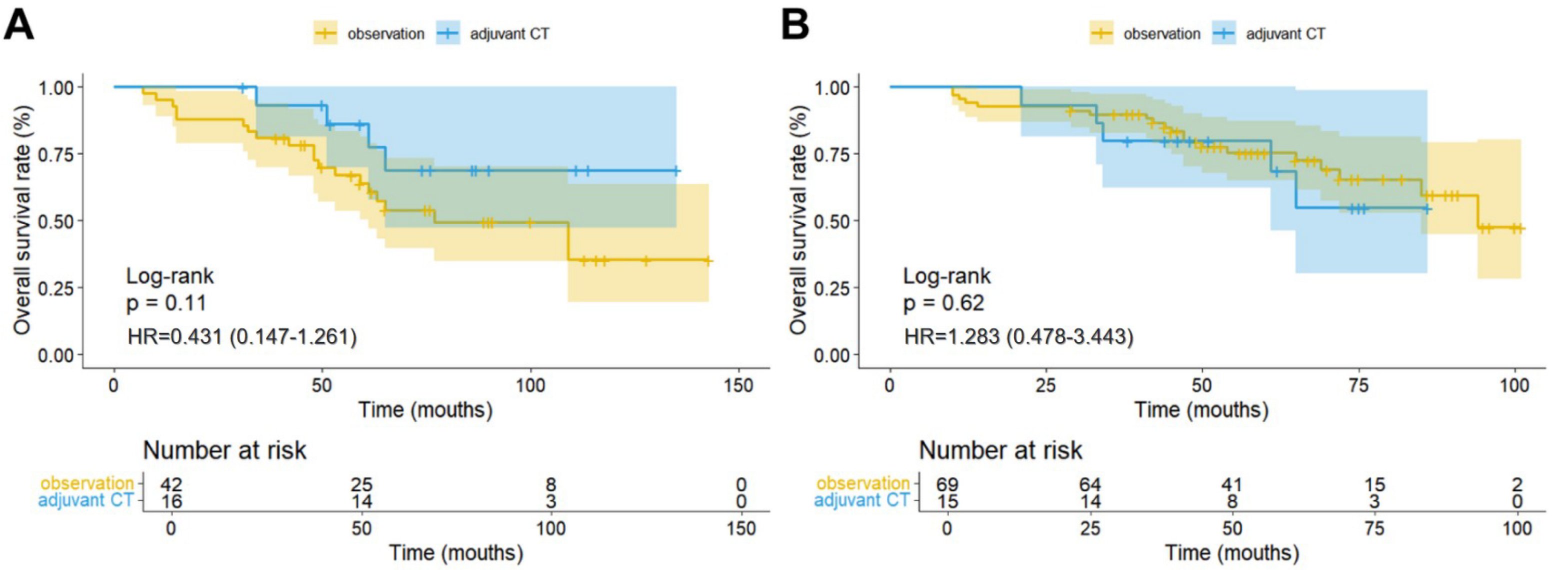

Supplementary Figure 2. Kaplan-Meier curves of the multicenter data for disease-free survival (A), overall survival (B) in patients with IASLC grade 3, disease-free survival (C) , overall survival (D) in patients with solid/micropapillary subtypes and disease-free survival (E), overall survival (F) in patients with VPI. CT, chemotherapy. IASLC, International Association for the Study of Lung Cancer ; VPI, visceral pleural infiltration.

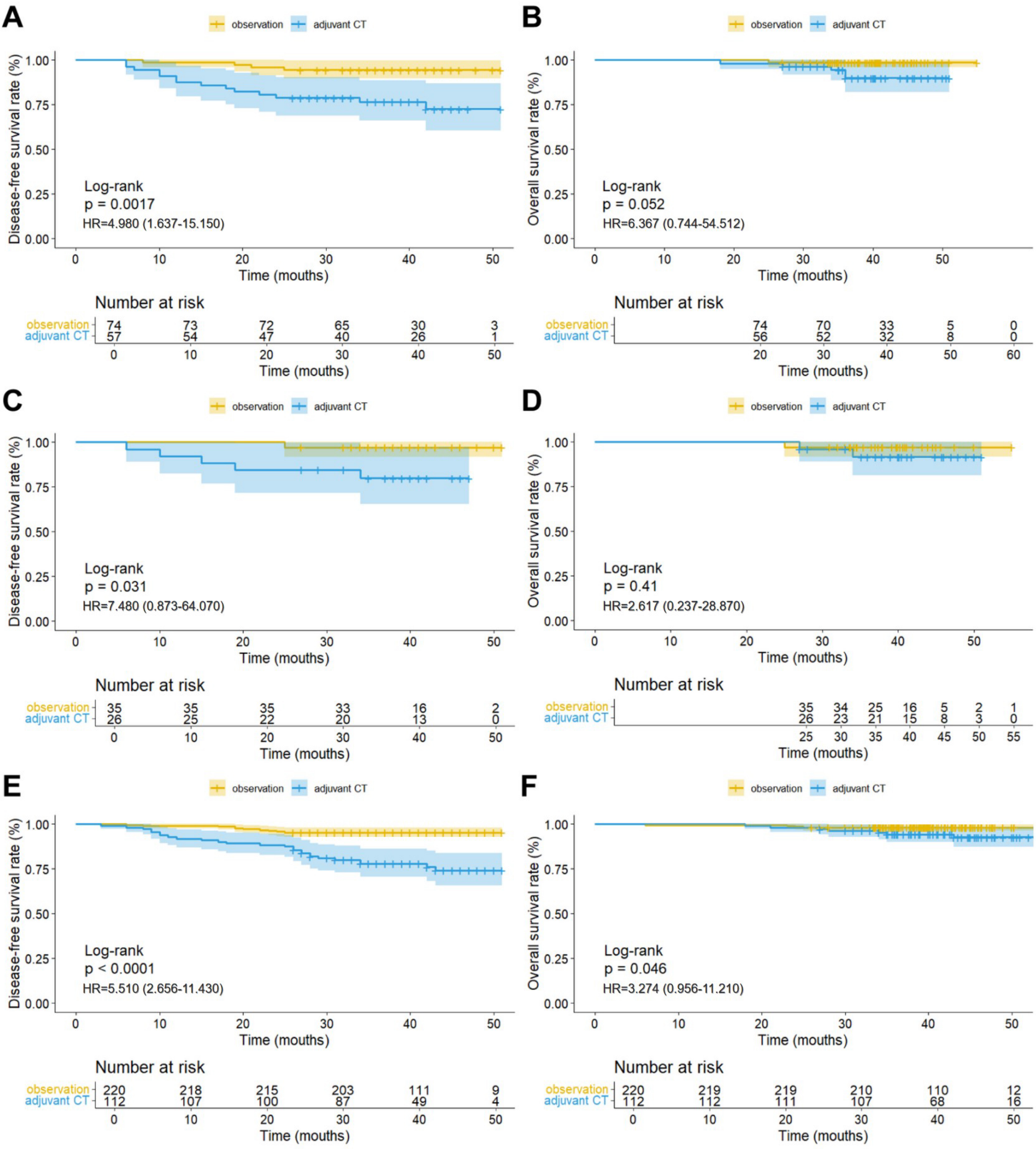

Supplement: Supplementary file 1 — Supplementary Material 1 [file 12957_2025_3907_MOESM1_ESM.pdf]
