# Supplementary material for: Limited benefit of adjuvant chemotherapy for Asian patients with stage IB lung adenocarcinoma: implications for clinical practice
Source: World J Surg Oncol. 2025 Jul 7;23:267. doi: 10.1186/s12957-025-03907-x (PMC12236002; doi:10.1186/s12957-025-03907-x)
Supplement: Supplementary file 2 — Supplementary Material 2 [file 12957_2025_3907_MOESM2_ESM.docx]

**Supplementary Table. 1 Baseline characteristics of propensity score matching between adjuvant chemotherapy and observation groups in non-Asian populations**

| **Characteristics** | **Before PSM** | | |  | **After PSM** | | |
| --- | --- | --- | --- | --- | --- | --- | --- |
|  | **Adjuvant CT**  **(n=406)** | **Observation**  **(n=2667)** | ***P* value** |  | **Adjuvant CT**  **(n=406)** | **Observation**  **(n=1524)** | ***P* value** |
| Sex, n (%)  Female  Male | 235 (57.9)  171 (42.1) | 1560(58.5)  1107 (41.5) | .816 |  | 235 (57.9)  171 (42.1) | 888 (58.3)  636 (41.7) | .889 |
| Age, n (%)  ≤60 y  >60 y | 177 (43.6)  229 (56.4) | 660 (24.7)  2007 (75.3) | <0.001 |  | 177 (43.6)  229 (56.4) | 591 (38.8)  933 (61.2) | .078 |
| Tumor size, n (%)  ≤3cm  >3cm | 178 (43.8)  228 (56.2) | 1380 (51.7)  1287 (48.3) | .003 |  | 178 (43.8)  228 (56.2) | 669 (43.9)  855 (56.1) | .984 |
| Laterality, n (%)  Left  Right | 156 (38.4)  250 (61.6) | 1042 (39.1)  1625 (60.9) | .804 |  | 250 (61.6)  156 (38.4) | 902 (59.2)  622 (40.8) | .383 |
| Grade, n (%)  Well and Moderately  Poorly  Undifferentiated | 248 (61.1)  148 (36.4)  10 (2.5) | 1835 68.8)  726 (27.2)  106 (4.0) | <0.001 |  | 248 (61.1)  148 (36.4)  10 (2.5) | 957 62.8)  496 (32.5)  71 (4.7) | .071 |
| VPI, n (%)  No  Yes  Undifferentiated | 89 (21.9)  79 (19.5)  238 (58.6) | 690 (25.9)  468 (17.5)  1509 (56.6) | .207 |  | 89 (21.9)  79 (19.5)  238 (58.6) | 366 (24.0)  256 (16.8)  902 (59.2) | .383 |
| LNs examined, n (%)  1-3  4-6  >6 | 67 (16.5)  99 (24.4)  240 (59.1) | 399 (15.0)  627 (23.5)  1641 (61.5) | .606 |  | 67 (16.5)  99 (24.4)  240 (59.1) | 277 (18.2)  385 (25.3)  862 (56.6) | .616 |

*PSM*, propensity score matching; *CT*, chemotherapy; *VPI*, visceral pleural infiltration; *LN*, lymph node.

**Supplementary Table. 2 Baseline characteristics of propensity score matching between adjuvant chemotherapy and observation groups in Asian populations**

| **Characteristics** | **Before PSM** | | |  | **After PSM** | | |
| --- | --- | --- | --- | --- | --- | --- | --- |
|  | **Adjuvant CT**  **(n=47)** | **Observation**  **(n=288)** | ***P* value** |  | **Adjuvant CT**  **(n=44)** | **Observation**  **(n=148)** | ***P* value** |
| Sex, n (%)  Female  Male | 34 (72.3)  13 (27.7) | 163(56.6)  125 (43.4) | .042 |  | 31 (70.5)  13 (29.5) | 98 (66.2)  50 (33.8) | .599 |
| Age, n (%)  ≤60 y  >60 y | 16 (34.0)  31 (66.0) | 63 (21.9)  225 (78.1) | .068 |  | 13 (29.5)  31 (70.5) | 34 (23.0)  114 (77.0) | .373 |
| Tumor size, n (%)  ≤3cm  >3cm | 26 (55.3)  21 (44.7) | 138 (47.9)  150 (52.1) | .347 |  | 25 (56.8)  19 (43.2) | 86 (58.1)  62 (41.9) | .879 |
| Laterality, n (%)  Left  Right | 23 (48.9)  24 (51.1) | 115 (39.9)  173 (60.1) | .245 |  | 23 (52.3)  21 (47.7) | 80 (54.1)  68 (45.9) | .835 |
| Grade, n (%)  Well and Moderately  Poorly  Undifferentiated | 31 (66.0)  16 (34.0)  0 (0.0) | 232 (80.5)  42 (14.6)  14 (4.9) | .002 |  | 31 (70.5)  13 (29.5)  0 (0.0) | 114 (77.0)  26 (17.6)  8 (5.4) | .083 |
| VPI, n (%)  No  Yes  Undifferentiated | 4 (8.5)  15 (31.9)  28 (59.6) | 77 (26.7)  69 (24.0)  142 (49.3) | .025 |  | 4 (9.1)  14 (31.8)  26 (59.1) | 19 (12.8)  42 (28.4)  87 (58.8) | .765 |
| LNs examined, n (%)  1-3  4-6  >6 | 7 (14.9)  10 (21.3)  30 (64.8) | 53 (18.4)  75 (26.0)  160 (55.6) | .569 |  | 7 (15.9)  10 (22.7)  27 (61.4) | 24 (16.2)  41 (27.7)  83 (56.1) | .785 |

*PSM*, propensity score matching; *CT*, chemotherapy; *VPI*, visceral pleural infiltration; *LN*, lymph node.

**Supplementary Table. 3 Baseline characteristics of propensity score matching between adjuvant chemotherapy and observation groups in multicenter populations**

| **Characteristics** | **Before PSM** | | |  | **After PSM** | | | | | | |
| --- | --- | --- | --- | --- | --- | --- | --- | --- | --- | --- | --- |
|  | **Adjuvant CT**  **(n=245)** | **Observation**  **(n=445)** | ***P* value** |  | | **Adjuvant CT**  **(n=200)** | **Observation**  **(n=200)** | | ***P* value** | |  |
| Sex, n (%)  Female  Male | 122 (49.8)  123 (50.2) | 244 (54.8)  201 (45.2) | .205 |  | 106 (53.0)  94 (47.0) | | | 104 (52.0)  96 (48.0) | | .841 | |
| Age , n (%)  ≤60y  >60y | 116 (47.3)  129 (52.7) | 166 (37.3)  279 (62.7) | .010 |  | 83 (41.5)  117 (58.5) | | | 82 (41.0)  118 (59.0) | | .919 | |
| Smoking history, n (%)  No  Yes | 170 (69.4)  75 (30.6) | 352 (79.1)  93 (20.9) | .004 |  | 151 (75.5)  49 (24.5) | | | 146 (73.0)  54 (27.0) | | .567 | |
| Tumor diameter, n (%)  ≤3cm  >3cm | 80 (32.7)  165 (67.3) | 176(39.6)  269 (60.4) | .073 |  | 63 (31.5)  137 (68.5) | | | 65 (32.5)  135 (67.5) | | .830 | |
| GGN, n (%)  No  Yes | 163 (66.5)  82 (33.5) | 271 (60.9)  174 (39.1) | .143 |  | 132 (66.0)  68 (34.0) | | | 131 (65.5)  69 (34.5) | | .916 | |
| Solid/micropapillary subtype, n (%)  No  Yes | 219 (89.4)  26 (10.6) | 410 (92.1)  35 (7.9) | .224 |  | 179 (89.5)  21 (10.5) | | | 181 (90.5)  19 (9.5) | | .739 | |
| IASLC grade, n (%)  Grade 1  Grade 2  Grade 3 | 23 (9.4)  165 (67.3)  57 (23.3) | 38 (8.5)  333 (74.8)  74 (16.6) | .081 |  | 16 (8.0)  143 (71.5)  41 (20.5) | | | 12 (6.0)  147 (73.5)  41 (20.5) | | .731 | |
| VPI, n (%)  No  Yes | 133 (54.3)  112 (45.7) | 225 (50.6)  220 (49.4) | .349 |  | 116 (58.0)  84 (42.0) | | | 114 (57.0)  86 (43.0) | | .840 | |

*PSM*, propensity score matching; *CT*, chemotherapy; *GGN*, ground-glass nodul*e*; *IASLC*, International Association for the Study of Lung Cancer ; *VPI*, visceral pleural infiltration.

**Supplementary Table. 4 Univariate and multivariate Cox regression analysis of OS in the non-Asian population**

| **Characteristics** | **Univariate Analysis** | |  | **Multivariate Analysis** | |
| --- | --- | --- | --- | --- | --- |
|  | **HR (95% CI)** | **p** |  | **HR (95% CI)** | **p** |
| Sex  Female  Male | 1  1.349 (1.206-1.510) | <0.001 |  | 1  1.361 (1.216-1.524) | <0.001 |
| Age (years)  ≤60  >60 | 1  1.921 (1.663-2.220) | <0.001 |  | 1  1.931 (1.667-2.235) | <0.001 |
| Tumor diameter (cm)  ≤3cm  >3cm | 1  1.125 (1.006-1.258) | 0.040 |  | 1  1.172 (1.040-1.321) | 0.009 |
| Laterality  Left  Right | 1  0.994 (0.886-1.115) | 0.923 |  |  |  |
| Grade  Well and Moderately  Poorly  Undifferentiated | 1  1.163 (1.029-1.315)  0.835 (0.595-1.171) | 0.016  0.295 |  | 1  1.240 (1.095-1.404)  0.877 (0.625-1.231) | 0.001  0.448 |
| VPI  No  Yes  Undifferentiated | 1  1.031 (0.843-1.261)  1.199 (1.033-1.392) | 0.764  0.017 |  | 1  1.136 (0.918-1.405)  1.272 (1.090-1.484) | 0.242  0.002 |
| LNs examined, n  1-3  4-6  >6 | 1  0.9019 (0.772-1.094)  0.798 (0.685-0.929) | 0.342  0.004 |  | 1  0.915 (0.769-1.090)  0.789 (0.677-0.919) | 0.320  0.002 |
| Adjuvant chemotherapy  No  Yes | 1  0.640 (0.530-0.773) | <0.001 |  | 1  0.695 (0.580-0.834) | <0.001 |

**Supplementary Table. 5 Univariate and multivariate Cox regression analysis of OS in the Asian population**

| **Characteristics** | **Univariate Analysis** | |  | **Multivariate Analysis** | |
| --- | --- | --- | --- | --- | --- |
|  | **HR (95% CI)** | **p** |  | **HR (95% CI)** | **p** |
| Sex  Female  Male | 1  1.581 (1.082-2.310) | 0.018 |  | 1  1.533 (1.047-2.245) | 0.028 |
| Age (years)  ≤60  >60 | 1  1.762 (1.036-2.998) | 0.037 |  | 1  1.694 (0.993-2.889) | 0.053 |
| Tumor diameter (cm)  ≤3cm  >3cm | 1  0.779 (0.532-1.139) | 0.198 |  |  |  |
| Laterality  Left  Right | 1  0.872 (0.595-1.277) | 0.481 |  |  |  |
| Grade  Well and Moderately  Poorly  Undifferentiated | 1  1.472 (0.933-2.321)  0.229 (0.032-1.647) | 0.096  0.143 |  |  |  |
| VPI  No  Yes  Undifferentiated | 1  1.346(0.747-2.425)  1.036 (0.611-1.722) | 0.322  0.923 |  |  |  |
| LNs examined, n  1-3  4-6  >6 | 1  0.716 (0.401-1.276)  0.922 (0.571-1.492) | 0.257  0.742 |  |  |  |
| Adjuvant chemotherapy  No  Yes | 1  0.799 (0.438-1.456) | 0.463 |  | 1  0.913 (0.498-1.674) | 0.768 |

**Supplementary Table. 6 Univariate and multivariate Cox regression analysis of DFS in the multi-center cohort**

| **Characteristics** | **Univariate Analysis** | |  | **Multivariate Analysis** | |
| --- | --- | --- | --- | --- | --- |
|  | **HR (95% CI)** | **p** |  | **HR (95% CI)** | **p** |
| Sex  Female  Male | 1  1.372 (0.852-2.212) | 0.194 |  |  |  |
| Age (years)  ≤60  >60 | 1  0.719(0.447-1.157) | 0.174 |  |  |  |
| Smoking history  No  Yes | 1  2.580 (1.598-4.165) | <0.001 |  | 1  2.150 (1.328-3.481) | 0.002 |
| Tumor diameter (cm)  ≤3cm  >3cm | 1  1.153 (0.698-1.906) | 0.579 |  |  |  |
| GGN  No  Yes | 1  0.386 (0.211-0.707) | 0.002 |  | 1  0.419 (0.229-0.767) | 0.005 |
| Solid/micropapillary subtype  No  Yes | 1  1.019 (0.441-2.355) | 0.965 |  |  |  |
| IASLC Grade 3  No  Yes | 1  1.644 (0.959-2.819) | 0.071 |  |  |  |
| VPI  No  Yes | 1  1.230 (0.764-1.981) | 0.394 |  |  |  |
| Adjuvant chemotherapy  No  Yes | 1  4.060 (2.442-6.750) | <0.001 |  | 1  3.679 (2.207-6.133) | <0.001 |

**Supplementary Table. 7 Univariate and multivariate Cox regression analysis of OS in Asian population with poorly differentiated in SEER database**

| **Characteristics** | **Univariate Analysis** | |  | **Multivariate Analysis** | |
| --- | --- | --- | --- | --- | --- |
|  | **HR (95% CI)** | **p** |  | **HR (95% CI)** | **p** |
| Sex  Female  Male | 1  1.786 (0.707-4.513) | 0.220 |  |  |  |
| Age (years)  ≤60  >60 | 1  1.493 (0.444-5.019) | 0.517 |  |  |  |
| Tumor diameter (cm)  ≤3cm  >3cm | 1  0.624 (0.277-1.405) | 0.255 |  | 1  0.530 (0.213-1.314) | 0.170 |
| Laterality  Left  Right | 1  0.977 (0.427-2.234) | 0.956 |  |  |  |
| VPI  No  Yes  Undifferentiated | 1  0.860(0.277-2.671)  0.622 (0.225-1.717) | 0.794  0.359 |  | 1  1.126 (0.318-3.992)  0.601 (0.189-1.911) | 0.854  0.388 |
| LNs examined, n  1-3  4-6  >6 | 1  0.502 (0.158-1.592)  0.331 (0.113-0.972) | 0.241  0.044 |  | 1  0.544 (0.161-1.834)  0.351 (0.117-1.055) | 0.326  0.062 |
| Adjuvant chemotherapy  No  Yes | 1  0.433 (0.148-1.268) | 0.127 |  | 1  0.478 (0.153-1.490) | 0.203 |

**Supplementary Table. 8 Univariate and multivariate Cox regression analysis of OS in Asian population with VPI in SEER database**

| **Characteristics** | **Univariate Analysis** | |  | **Multivariate Analysis** | |
| --- | --- | --- | --- | --- | --- |
|  | **HR (95% CI)** | **p** |  | **HR (95% CI)** | **p** |
| Sex  Female  Male | 1  1.162 (0.518-2.607) | 0.715 |  |  |  |
| Age (years)  ≤60  >60 | 1  1.649 (0.561-4.849) | 0.363 |  |  |  |
| Tumor diameter (cm)  ≤3cm  >3cm | 1  0.537 (0.213-1.351) | 0.186 |  | 1  0.480 (0.180-1.279) | 0.142 |
| Laterality  Left  Right | 1  1.072 (0.487-2.356) | 0.863 |  |  |  |
| Grade  Well and Moderately  Poorly  Undifferentiated | 1  1.373 (0.544-3.467)  N/A | 0.502  N/A |  | 1  1.714 (0.583-5.040)  N/A | 0.327  N/A |
| LNs examined, n  1-3  4-6  >6 | 1  1.107 (0.321-3.816)  1.092 (0.356-3.346) | 0.871  0.877 |  | 1  1.025 (0.281-3.734)  0.904 (0.290-2.817) | 0.970  0.862 |
| Adjuvant chemotherapy  No  Yes | 1  1.281 (0.477-3.436) | 0.623 |  | 1  1.015 (0.345-2.983) | 0.979 |

**Supplementary Table. 9 Univariate and multivariate Cox regression analysis of DFS in patients with IASLC grade 3 in multicenter data**

| **Characteristics** | **Univariate Analysis** | |  | **Multivariate Analysis** | |
| --- | --- | --- | --- | --- | --- |
|  | **HR (95% CI)** | **p** |  | **HR (95% CI)** | **p** |
| Sex  Female  Male | 1  0.605 (0.240-1.525) | 0.287 |  |  |  |
| Age (years)  ≤60  >60 | 1  0.402 (0.156-1.039) | 0.060 |  |  |  |
| Smoking history  No  Yes | 1  0.972 (0.365-2.589) | 0.954 |  |  |  |
| Tumor diameter (cm)  ≤3cm  >3cm | 1  1.592 (0.568-14.467) | 0.377 |  | 1  2.567 (0.722-9.123) | 0.145 |
| GGN  No  Yes | 1  0.579 (0.168-2.000) | 0.388 |  | 1  0.584 (0.166-2.054) | 0.584 |
| Solid/micropapillary subtype  No  Yes | 1  0.543 (0.204-1.448) | 0.223 |  | 1  0.454 (0.164-1.258) | 0.129 |
| VPI  No  Yes | 1  1.163 (0.459-2.946) | 0.751 |  | 1  2.482 (0.793-7.762) | 0.118 |
| Adjuvant chemotherapy  No  Yes | 1  4.963 (1.631-15.101) | 0.005 |  | 1  4.749 (1.543-14.614) | 0.007 |

**Supplementary Table. 10 Univariate and multivariate Cox regression analysis of DFS in patients with VPI in multicenter data**

| **Characteristics** | **Univariate Analysis** | |  | **Multivariate Analysis** | |
| --- | --- | --- | --- | --- | --- |
|  | **HR (95% CI)** | **p** |  | **HR (95% CI)** | **p** |
| Sex  Female  Male | 1  1.869 (0.968-3.609) | 0.063 |  |  |  |
| Age (years)  ≤60  >60 | 1  0.636 (0.330-1.228) | 0.178 |  |  |  |
| Smoking history  No  Yes | 1  3.078 (1.586-5.974) | 0.001 |  | 1  2.640 (1.342-5.193) | 0.005 |
| Tumor diameter (cm)  ≤3cm  >3cm | 1  1.935 (0.990-3.783) | 0.053 |  | 1  1.566 (0.798-3.072) | 0.193 |
| GGN  No  Yes | 1  0.521 (0.245-1.108) | 0.090 |  | 1  0.626 (0.292-1.341) | 0.228 |
| Solid/micropapillary subtype  No  Yes | 1  1.544 (0.600-3.971) | 0.368 |  | 1  0.637 (0.182-2.229) | 0.480 |
| IASLC Grade 3  No  Yes | 1  1.596 (0.769-3.311) | 0.209 |  | 1  1.448 (0.554-3.785) | 0.450 |
| Adjuvant chemotherapy  No  Yes | 1  5.498 (2.650-11.406) | <0.001 |  | 1  4.947 (2.376-10.302) | <0.001 |

**Supplementary Table. 11 Univariate and multivariate Cox regression analysis of DFS in patients with solid/micropapillary subtypes in multicenter data**

| **Characteristics** | **Univariate Analysis** | |  | **Multivariate Analysis** | |
| --- | --- | --- | --- | --- | --- |
|  | **HR (95% CI)** | **p** |  | **HR (95% CI)** | **p** |
| Sex  Female  Male | 1  0.659 (0.121-3.598) | 0.630 |  |  |  |
| Age (years)  ≤60  >60 | 1  0.846 (0.155-4.618) | 0.847 |  |  |  |
| Smoking history  No  Yes | 1  1.514 (0.305-7.510) | 0.612 |  |  |  |
| Tumor diameter(cm)  ≤3cm  >3cm | 1  0.881 (0.161-4.809) | 0.883 |  | 1  1.921 (0.326-11.318) | 0.471 |
| GGN  No  Yes | 1  0.537 (0.063-4.599) | 0.571 |  | 1  0.573 (0.066-4.985) | 0.614 |
| VPI  No  Yes | 1  4.483 (0.524-38.374) | 0.171 |  | 1  4.956 (0.530-46.337) | 0.161 |
| Adjuvant chemotherapy  No  Yes | 1  7.480 (0.873-64.074) | 0.066 |  | 1  6.658 (0.770-57.559) | 0.085 |

**Supplementary Table. 12 Univariate and multivariate Cox regression analysis of OS in patients with IASLC grade 3 in multicenter data**

| **Characteristics** | **Univariate Analysis** | |  | **Multivariate Analysis** | |
| --- | --- | --- | --- | --- | --- |
|  | **HR (95% CI)** | **p** |  | **HR (95% CI)** | **p** |
| Sex  Female  Male | 1  1.219 (0.223-6.657) | 0.819 |  |  |  |
| Age (years)  ≤60  >60 | 1  1.425 (0.261-7.783) | 0.682 |  |  |  |
| Smoking history  No  Yes | 1  4.095 (0.749-22.384) | 0.104 |  |  |  |
| Tumor diameter (cm)  ≤3cm  >3cm | 1  0.609 (0.123-3.018) | 0.544 |  | 1  0.905 (0.110-7.424) | 0.926 |
| GGN  No  Yes | 1  1.512 (0.277-8.254) | 0.633 |  | 1  1.479 (0.261-8.373) | 0.658 |
| Solid/micropapillary subtype  No  Yes | 1  1.154 (0.233-5.720) | 0.860 |  | 1  1.061 (0.202-5.586) | 0.944 |
| VPI  No  Yes | 1  1.936 (0.354-10.572) | 0.446 |  | 1  1.868 (0.203-17.222) | 0.581 |
| Adjuvant chemotherapy  No  Yes | 1  6.343 (0.741-54.299) | 0.092 |  | 1  6.589 (0.768-56.563) | 0.086 |

**Supplementary Table. 13 Univariate and multivariate Cox regression analysis of OS in patients with VPI in multicenter data**

| **Characteristics** | **Univariate Analysis** | |  | **Multivariate Analysis** | |
| --- | --- | --- | --- | --- | --- |
|  | **HR (95% CI)** | **p** |  | **HR (95% CI)** | **p** |
| Sex  Female  Male | 1  1.747 (0.533-5.727) | 0.357 |  |  |  |
| Age (years)  ≤60  >60 | 1  2.258 (0.599-8.512) | 0.229 |  |  |  |
| Smoking history  No  Yes | 1  4.857 (1.482-15.918) | 0.009 |  | 1  4.066 (1.200-13.783) | 0.024 |
| Tumor diameter (cm)  ≤3cm  >3cm | 1  1.692 (0.495-5.781) | 0.401 |  | 1  1.352 (0.391-4.676) | 0.634 |
| GGN  No  Yes | 1  0.924 (0.270-3.156) | 0.899 |  | 1  1.349 (0.377-4.824) | 0.645 |
| Solid/micropapillary subtype  No  Yes | 1  3.566 (0.945-13.458) | 0.061 |  | 1  2.160 (0.217-21.476) | 0.511 |
| IASLC Grade 3  No  Yes | 1  2.283 (0.668-7.798) | 0.188 |  | 1  1.035 (0.126-8.506) | 0.974 |
| Adjuvant chemotherapy  No  Yes | 1  3.274 (0.956-11.212) | 0.059 |  | 1  2.794 (0.805-9.700) | 0.106 |

**Supplementary Table. 14 Univariate and multivariate Cox regression analysis of OS in patients with solid/micropapillary subtypes in multicenter data**

| **Characteristics** | **Univariate Analysis** | |  | **Multivariate Analysis** | |
| --- | --- | --- | --- | --- | --- |
|  | **HR (95% CI)** | **p** |  | **HR (95% CI)** | **p** |
| Sex  Female  Male | 1  0.650 (0.059-7.170) | 0.725 |  |  |  |
| Age (years)  ≤60  >60 | 1  0.906 (0.082-10.010) | 0.935 |  |  |  |
| Smoking history  No  Yes | 1  3.234 (0.292-35.838) | 0.339 |  |  |  |
| Tumor diameter (cm)  ≤3cm  >3cm | 1  0.204 (0.018-2.255) | 0.195 |  | 1  0.232 (0.020-2.682) | 0.242 |
| GGN  No  Yes | 1  1.394 (0.126-15.386) | 0.786 |  | 1  1.465 (0.133-16.170) | 0.755 |
| VPI  No  Yes | N/A  N/A | N/A  N/A |  |  |  |
| Adjuvant chemotherapy  No  Yes | 1  2.617 (0.237-28.868) | 0.432 |  | 1  2.009 (0.174-23.236) | 0.576 |

-
